# Taxonomy of the Family Teneriffiidae (Acari: Prostigmata: Anystoidea): Generic Synonymies with the Key to World Species of the Family

**DOI:** 10.3390/ani13233736

**Published:** 2023-12-02

**Authors:** Jawwad Hassan Mirza, Muhammad Kamran, Fahad Jaber Alatawi

**Affiliations:** Department of Plant Protection, College of Food and Agriculture Sciences, King Saud University, Riyadh 11451, Saudi Arabia; jmirza@ksu.edu.sa (J.H.M.); murafique@ksu.edu.sa (M.K.)

**Keywords:** palpgenu oncophysis, species synonymy, history, scientific gaps, literature review, character strength, distribution

## Abstract

**Simple Summary:**

The generic divisions of the family Teneriffiidae have been dealt with superficially, by which different morphological features were introduced over time to justify the addition of apparently unnecessary genera. The present research provides thorough and detailed insight into the taxonomy of the family Teneriffiidae, and different morphological characters were evaluated. As a result, two genera, *Teneriffia* Thor and *Parateneriffia* Thor, were considered valid based on persistent morphological character/s. The other existing genera were synonymized, and a diagnostic key to genera and species of the family Teneriffiidae was developed while four species were synonymized.

**Abstract:**

The family Teneriffiidae Thor has an equivocal and patchy generic history due to a lack of proper diagnostic character/s, causing the addition of an over-sufficient number of genera (i.e., nine) for the 28 described species. The present study aimed to resolve those taxonomic uncertainties related to generic divisions and species assignments by thoroughly reviewing all the published literature of the family, identifying key diagnostic character/s for generic divisions while debating on previously used morphological features. In the present research, only two genera, *Teneriffia* Thor and *Parateneriffia* Thor, are considered valid genera in the family Teneriffiidae, based on the absence and presence of palpgenu oncophysis, respectively. The previously used other generic diagnostic characters such as coxal setal formula, pectination strength of leg claws, absence or presence of genital papillae, genital discs, and pedal solenidion have been argued for their inconsistencies. A total of four species were synonymized with the closely related species, while additional notes for six poorly described species are given. Moreover, the key to the genera and species of the family Teneriffiidae is provided.

## 1. Introduction

The members of the family Teneriffiidae Thor (Acari: Prostigmata: Anystoidea) are moderate-sized fast-walking mites, usually found in terrestrial (trees, rocks, caves, mountains, etc.) and occasionally in marine habitats [1,2,3]. They are predatory, feeding on small arthropods [1,4]. After hatching from eggs, individuals undergo four immature stages, including larva, protonymph, deutonymph, and tritonymph, before molting into adults [5]. The biology and ecology of teneriffids are poorly studied, with a single observation of an immobile pre larva enclosed in an eggshell [6]. 

The diagnostic morphological features of the family Teneriffiidae include the presence of bothridial setae on prodorsum with a rosette-patterned base, disc-like palp tarsus, strong and simple palp tibial claw, subtended by two smaller, straight spurs, oncophysis on palpgenu absent or present, strongly bipectinated claws of at least leg I, and claw-like empodium present on legs III–IV [4]. Currently, there are about 28 globally reported species belonging to nine genera [3]. However, these figures have been contrastingly reported in some recent publications [2,3,7,8]. 

For the number of described species in the family Teneriffiidae, the number of genera erected has been previously questioned [9,10]. As a result of this, different taxonomic revisions were made where genera were either synonymized or reinstated [5,9,10,11,12,13,14,15]. Some useful taxonomic information was presented by McDaniel et al. [10], Judson [13,14], Schmölzer [15], and Ueckermann et al. [16]. 

Even after these revisions, the comprehensive literature review of the family Teneriffiidae has shown that the taxonomic history of its genera have scientific uncertainties and research gaps due to different reasons, including lost type specimens, generic additions or revisions with missing references, overlooked valid species, immatures being considered as adults, etc. This has led to the dire need for significant taxonomic revision of the family where all species and their assigned genera must be re-evaluated based on distinct and persistent morphological characters. The aims of the present study were to highlight and resolve scientific uncertainties related to generic divisions and species assignments in the family Teneriffiidae by assessing the previously defined genera and species and identifying key diagnostic character/s for generic divisions while debating on previously used morphological features of generic division. A diagnostic key to the genera and reported species of the family Teneriffiidae is also provided.

## 2. Materials and Methods

The taxonomic literature of all nine genera and 28 teneriffid species were critically studied, and the diagnostic characters of the genera were compared. For the differentiation among different developmental stages, McDaniel et al. [10] and Judson [13] were followed. The tables for comparative morphologies and addition of genera and species over time were constructed based on the available published literature. The strength of each morphological character was evaluated for its suitability at the generic level. The key to species of the family Teneriffiidae is provided based on persistent and fixed characteristics.

## 3. Historical Background of the Family Tenerifiidae

The family Teneriffiidae was erected by Thor in 1911 with two monotypic genera: *Teneriffia* Thor (type genus; type species *T. quadrapapillata*) and *Parateneriffia* Thor (type species *P. bipectinata*) [17] (Table 1). In 1924, Hirst erected the third genus *Neoteneriffiola* (type species *N. luxoriensis*) [18], while the fourth genus *Heteroteneriffia* (type species *H. marina*) was added in 1925 [19]. All four genera were distinguished based on a number of coxal setae I–IV, state of coxal segments, presence or absence of oncophysis on palp genu, and strength of pectination on claws of legs I–IV (Table 1 and Table 2).

In 1935, Womersley [20] added the fifth genus *Austroteneriffia* (type species *A. hirsti*) and considered it closely related to the genus *Heteroteneriffia* based on the presence of genital discs (papillae) (absent in *Teneriffia* and *Neoteneriffiola* genera), the differing claws of leg I–II which strongly pectinated (on only leg I in *Heteroteneriffia*), and not having a definite row of setae on anterior margins of the coxae in *Austroteneriffia* (Table 1).

Later, two more monotypic genera (sixth and seventh in series) were added to the family, namely *Mesoteneriffia* Irk [21] (type species *M. steinbocki*) and *Mesoteneriffiola* Schmölzer [22] (type species *M. alpina*). The genus *Mesoteneriffia* was considered close to the genus *Parateneriffia* due to the presence of palpgenu oncophysis and all leg coxae lying close together. These two were separated due to the absence of a genital clasping organ in *Mesoteneriffia*. The genus *Mesoteneriffiola* was separated from *Mesoteneriffia* mainly based on the number of setae on coxae I–IV (4-4-4-4 vs. 3-3-3-1) (Table 1).

At this point, the overwhelming number of genera for the number of species described (seven genera for eight species) was first time criticized, but no work on generic revision was performed [9] (Table 2). Later, new synonymies were proposed, recognizing only two valid genera in the family Teneriffiidae, i.e., *Teneriffia* (genus *Heteroteneriffia* synonymized) and *Parateneriffia* (three genera; *Neoteneriffiola*, *Austroteneriffia*, and *Mesoteneriffiola* synonymized) [10] (Table 1).

The genus *Neoteneriffiola* (third after McDaniel et al. [10]) was later reinstated, while the previous synonymy was criticized, stating the reasons as lack of paratype observation and inadequate original description of *Parateneriffia* [13]. Simultaneously, a unique species of the reinstated *Neoteneriffiola* genus was reported, and its significance for the basis of a new genus was highlighted, although none was added.

In the same year, another genus (eighth in series and fourth after McDaniel et al. [10]), *Sinoteneriffia*, was added to the family [23]. This genus was separated from *Neoteneriffiola* based on the number of coxal setae, the number of setae on and around the gential valve, and the number of reproductive suckers. 

After almost a year, the types of the *Austroteneriffia* genus were revisited [14] (Table 1) and declared as a valid genus (fifth after McDaniel et al. [10]). Also, some species from the previously reinstated *Neoteneriffiola* genus were transferred to the reinstated genus *Austroteneriffia* [14].

The genus *Himalteneriffia* (type species; *H. riccabonai*) (ninth in series, sixth after McDaniel et al. [10]) was added to the family Teneriffiidae [15]. While defining the genus *Himalteneriffia*, different morphological and geographical aspects of only 8 genera (*Teneriffia*, *Parateneriffia*, *Austroteneriffia*, *Mesoteneriffia*, *Mesoteneriffiola*, *Heteroteneriffia* and *Himalteneriffia*) and 14 species of the family Teneriffiidae were studied [15]. After critically evaluating all the published literature on the family Teneriffiidae, it was found in the present study that there are six genera (*Austroteneriffia*, *Himalteneriffia*, *Neoteneriffiola*, *Parateneriffia*, *Sinoteneriffia* and *Teneriffia*) reported in the family Teneriffiidae which were either originally described or reinstated after McDaniel et al. [10]. These genera are comprised of eleven, two, five, one, two, and seven species, respectively. The status of the genus *Mesoteneriffiola* (and its species *M. alpina*) after McDaniel et al. [10] is still unknown and will be discussed.

## 4. Results

### 4.1. Taxonomic Uncertainties and Scientific Gaps in the Literature

Throughout the systematic journey of the family Teneriffiidae, its genera were dealt with superficially, and unstable features were used to erect the teneriffid genera. This has resulted in an overall confused taxonomic perspective towards the strength and reliability of morphological characters to be either used for the generic or species level. This will all be discussed in chronological order, where different taxonomic uncertainties and scientific gaps will be highlighted.

McDaniel et al. [10], for the first time, proposed generic synonymies and an in-depth review in the present research work, highlighting the following five shortcomings:

(i) The important published literature, prior to/close to 1976, was not considered and this concern was also previously raised [14]. The revised diagnoses of *Neoteneriffiola* and *Heteroteneriffia* by Ehara [11] and of *Austroteneriffia* by Shiba and Furukawa [5] were not cited. Due to this, one of the incorrect arguments raised by these authors for the synonymy of *Austroteneriffia* with *Parateneriffia* was stated as “*Also, A. hirsti is terrestrial in habit similar to the Parateneriffia-Neoteneriffiola complex whereas the Teneriffia-Heteroteneriffia complex is littoral*”. The authors would not have made this statement if the species, *A. littorina*, reported as littoral, ref. [5] was considered.
(ii) The palpgenu oncophysis was reported missing from the genus *Austroteneriffia* based on the description of species, *A. hirsti*. However, Judson [14] reported the presence of palpgenu oncophysis (the “distal process”) in the redescription of *A. hirsti* after observing the type specimens. It further contributes to weakening the proposed synonymy.
(iii) While synonymizing the genus *Neoteneriffiola*, three described species (*N. japonica* Ehara, *N. tadjikistanica* Wainstein, and *N. hojoensis* Shiba and Furukawa) were excluded from the work. This makes the status of these species uncertain.
(iv) The character of coxal setal counts was used in a very general manner while bringing *Austroteneriffia* (i.e., some coxae have 4 or fewer setae) and *Mesoteneriffia* (i.e., only four setae on coxae) close to *Parateneriffia*-*Neoteneriffiola* complex. This is not true, particularly for *Parateneriffia*, in which coxae III has seven setae as described and illustrated in original work [17] and ironically reported by the authors in the key [10].
(v) Another monotypic genus *Mesoteneriffiola*, which was reported close to *Mesoteneriffia* was not even mentioned during this review. The validity of this genus was uncertain as only two valid genera were recognized, i.e., *Teneriffia* and *Parateneriffia*.

During the reinstatement of the genera *Neoteneriffiola* [13] and *Austroteneriffia* [14], morphology-based comparisons were not provided and it was left for the readers to figure out the diagnostic characters of these reinstated genera. However, based on the emended diagnosis, the characters which could be considered distinguishing for the genus *Austroteneriffia* were a low number of pedal solenidia and holotrichous aggenital chaetotaxy [14]. Interestingly, these characters were already present in the diagnosis of the previously reinstated genus *Neoteneriffiola*, (except the species, *N. coineaui*; neotrichy of pedal solenidia). This raises reservations on the overall generic reinstatement. Also, some species described in *Neoteneriffiola* were moved to the genus *Austroteneriffia* without the provision of compelling morphology-based remarks.

The new genus, *Himalteneriffia*, was added to the family Teneriffiidae [15] without citing the important previously published taxonomic literature. Not only a genus (*Sinoteneriffia*) was missed in the generic analysis, but the genera *Heteroteneriffia* and *Mesoteneriffia* were considered valid without any remarks after previous synonymies of McDaniel et al. [10] and the work of Judson [13]. Also, the previously described eight species were overlooked [15]. 

Also, it is important to mention that uncertainties can still be found in the recently published work of the family Teneriffiidae. Ueckermann and Durucan [7] mentioned there are eight genera in the family where *Heteroteneriffia* (three species) and *Mesoteneriffia* (two species) were added in the generic count while genus *Mesoteneriffiola* was excluded. Zumudzinski et al. [2] believed in the presence of about 20 species in 9 genera. These authors considered those three genera as valid. Paktinat-Saeij and Kazemi [8] also reported 27 species in 9 genera. It is worth mentioning that even though the genus *Heteroteneriffia* has been considered valid, the provided number of species is incorrect. Shiba and Furukawa [5] synonymized the species *T. tokiokai* (Ehara) with *T. marina* (Hirst). Lastly, Beron [3] provided the catalogue for the family Teneriffiidae. Although the correct number of species in each genus was provided, the author still considered the three genera as valid. 

As a result of the thorough literature review in the present study, it became evident that taxonomic ranks were treated sloppily in the family Teneriffiidae. Different morphological characters were used without measuring their taxonomic significance and the possibility of variability in the character states. The missing references in the published works and lack of comparative morphological analysis of genera and species has only further downgraded the situation. It is crucial to validate the significance of each character at different taxonomic ranks.

### 4.2. Strength of Morphological Characters for Generic Divisions

During 1911–1925, the genera were separated based on intercoxal distances, the presence or absence of palpgenus oncophysis, the number of setae on coxae I–IV, and pectination strength of leg tarsal claws (Table 1). Womersley [20] introduced the absence and presence of a gential disc and definite setal row on the anterior margin of coxae. Eller and Strandtmann [9] debated on the character of genital disks, attributing it as a sexual difference. Irk [21] again used the characters of palpgenu oncophysis, intercoxal distances, and further added chitinous process on coxa I and the presence or absence of bracket field (translated from original German description “*Vor der Genitalöffnung ein „Spangenfeld*”). McDaniel [10], while synonymizing the genera, considered the number of ventral opisthosomal setae, the presence or absence of palpgenu oncophysis, the length of legs comparative to body, and the number of setae on coxae I–IV. Judson [13,14], during the reinstatement of two genera, placed emphasis on the neotrichy of pedal solenidia, the size of dorsal opisthosomal shield and relatively large dorsal plates, and the reduced form of peritremes. Schmölzer [15] also considered dorsal shield size, the ridges on leg claws I–IV, and the number of setae on coxae I–IV as generic character.

Throughout the taxonomic history of adding, synonymizing, and reinstating the genera of the family Teneriffiidae, two morphological characters, i.e., palpgenus oncophysis and the number of setae on coxae I–IV, were found to be repeatedly used. The number of coxal setae appear unreliable as it has been reported to be variable not only among different populations of a species but even in one population of single species (Table 3). However, in two genera out of nine, *Austroteneriffia* (eleven species) and *Neoteneriffiola* (five species) this character is quiet stable among all the described species. On the other hand, the palpgenu oncophysis is a very persistent and stable character among all the described species and genera of the family Teneriffiidae, with only two states, i.e., present or absent. 

Interestingly, the character of pectination strength on leg claws appeared once to differentiate the genus *Heteroteneriffia*. However, this genus is still under synonymy with the genus *Teneriffia* [13]. The number of setae on ventral opisthosoma near genital region being numerous belong to two genera, i.e., *Heteroteneriffia* and *Teneriffia*. The character of genital discs, as mentioned earlier, cannot be used for generic differentiations as it differs between female and male [9]. In the present study, based on these two characters, the synonymy of *Heteroteneriffia* with *Teneriffia* is considered valid. 

After analyzing all the morphological characters ever used for the generic differentiation in the present study, it became suitable and convenient to place the finger on the most persistent morphological character, i.e., palpgenu oncophysis. This character is found in all the described stages and in both females and males, and it could be the most suitable for the generic divisions.

### 4.3. Generic Division

Among the 28 described species in the family Teneriffiidae, different species were described either from male or female or both (Table 4). The male descriptions and illustrations were provided for only 18 species (63%), while females are described and illustrated from all the species (100%). After the detailed study of the published literature of all teneriffid species, two genera, *Teneriffia* Thor and *Parateneriffia* Thor, are considered as valid in this study, for all the described teneriffid species based on the presence and absence of palpgenu oncophysis in females (Table 3). The genera *Heteroteneriffia*, *Himalteneriffia*, and *Sinoteneriffia* are hereby synonymized with the genus *Teneriffia* (absence of palpgenu oncophysis). The genera *Austroteneriffia*, *Neoteneriffiola*, *Mesoteneriffia* and *Mesoteneriffiola* are synonymized with the genus *Parateneriffia* (presence of palpgenus oncophysis). Out of the 28 species described up to now, 24 species are assigned between these two genera (excluding four proposed species synonymies).

**Family Teneriffiidae Thor**

Teneriffiidae Thor 1911:179 [17]

Teneriffiolidae Hirst, 1924: 1078 [18]

Teneriffiinae Womersley, 1935: 334 [20]

Type genus: *Teneriffia* Thor, 1911 [17]

**Diagnosis:**

The diagnosis of the family has been provided by several authors [4,9,20,25]. In the present study, a precisely updated family diagnosis is provided.

Naso present, small and without setae, prodorsal bothridial setae with rosette patterned base, palp tarsus reduced; disc like, palp tibial claw strong with two small spurs at the base, chelicerae with sickle like chelae, not fused proximally, pretarsal empodial claws absent on legs I–II while present on legs III–IV, the true claws on at least leg I highly pectinated, peritremes not emargant, multichambered and present anterolateraly.

**Genus *Teneriffia* Thor**

*Teneriffia* Thor 1911:172 [17]

*Heteroteneriffia* Hirst 1925:1278 [19]. Type species *H. marina* Hirst 1925 [19]

*Sinoteneriffia* Yin et al. 1994:443 [23]. Type species *S. nuda* Yin et al. 1994 [23]

*Himalteneriffia* Schmölzer 2002:133 [15]. Type species *H. riccabonai* Schmölzer 2002 [15]

**Type species**: by original designation, *T. quadripapillata*, Thor 1911:173 [17], Ueckermann et al. 2022: 789 [16].

**Diagnosis**: Palpgenu oncophysis absent, prodorsal shield either present or absent.

**Number of species included**: 10 (Table 3)

**Distribution**: Mexico, Spain, Turkey, Iran, Japan, India, Malaysia

**Remarks**: This genus is retained based on original designation by Thor [17] as type genus of the family. Ueckermann et al. [16] recollected a number of specimens from type locality. This genus was originally described with the palpgenu oncophysis absent which is endorsed in the present study. 

**Genus *Parateneriffia* Thor**

*Parateneriffia* Thor 1911:176 [17]

*Neoteneriffiola* Hirst 1924:1078 [18]. Type species *N. luxoriensis* Hirst 1924 [18]

*Austroteneriffia* Womersley 1935:334 [20]. Type species *A. hirsti* Womersley 1935 [20]

*Mesoteneriffia* Irk 1939:220 [21]. Type species *M. steinbocki* Irk 1939 [21]

*Mesoteneriffiola* Schmölzer 1955:36 [22]. Types species *M. alpina* Schmölzer 1955 [22]

**Type species**: *Parateneriffia bipectinata* Womersely

**Diagnosis**: palpgenu oncophysis present, prodorsal shield always present.

**Number of species included**: 14 (Table 3)

**Distribution**: Ethiopia, Namibia, Egypt, Mexico, USA, Brazil, China, Japan, Iran, Tadjikistan, Yemen, Australia, Paraguay, Austria, Switzerland, France

**Remarks**: This genus is retained based on its original designation by Thor [17] as the second genus in the family Teneriffiidae. It was originally diagnosed by the presence of palpgenu oncophysis, which is endorsed in the present study. The original type of the genus was *P. bipectinata* [17]. This species was criticized due to the loss of its type specimens and an inadequate original description and illustration [13].

### 4.4. On the Suggested Synonymy of Some Species

The species, *P. hojoensis* (Shiba and Furukawa) was originally distinguished from *P. japonica* (Ehara) based on the presence or absence of a solenidion on leg genu I–IV, i.e., leg genu I–IV solenidotaxy as 1-1-1-0 and 0-0-0-0, respectively [5,11]. Later, a short description of *A. japonica* reported the presence of solenidion on leg genu I–II [14]. Here, in this study, a critical review of the descriptions of both the species revealed a few differences as in leg chaetotaxy and solenidotaxy. Other than that, these two species are morphologically resembling. The species *P. japonica* was originally described from two males while *P. hojoensis* was originally described from more than ten individuals of male, female, deutonymph, and protonymph. Additionally, both species were reported from Japan. In the present study, these two species belong to the genus *Parateneriffia* (presence of palpgenu oncophysis). However, based on the argument provided above, *P. hojoensis* is suggested as junior synonym of *P. japonica*.

Youzhen et al. [27] described the species *Neoteneriffiola yunnanesis* based on the male, with few morphological characters which were typical of the genus. The original remarks placed this species close to *P. japonica* and *P. tadjikistanica* and differentiated it based on body length and number of setae on genu IV. Also, the original description did not include the *P. hojoensis* in the key. It became clear upon comparing the original descriptions of these three species that *N. yunnanensis* resembles *P. japonica* and *P. hojoensis* and it is suggested as synonym of *P. japonica*. 

There are two species described under the genus *Sinoteneriffia* by Yin et al. [23]. As argued earlier the genus and its type species *S. nuda* were diagnosed based on deutonymphal characters (genital shield without setae, two setae around genital shield) and hence are not valid. Similarly, the second species, *S. kunmingensis*, described by Youzhin et al. [26] was also diagnosed on the supposed male but has similar characters to the deutonymphal stage. Hence, the genus *Sinoteneriffia*, as stated above, and its two species are not valid because both species were described based on deutonymphs.

### 4.5. Key to Genera and Species of the Family Teneriffiidae Based on Females

1. Palpgenu oncophysis absent .................................. genus *Teneriffia* Thor .............................. 2- Palpgenus oncophysis present ................................ genus *Parateneriffia* Thor ...................... 112. Gnathosoma ventrally with clasp organ ................................................ *T. quadripapillata* Thor- Gnathosoma without ventral clasp organ .................................................................................. 33. Only claws of leg I heavily pectinated ...................................................................................... 4- Claws of leg I–II heavily pectinated ............................................................................................ 54. Naso punctate, spurs of hypostome elongate, tarsus II with three solenidia ........ ........................................................................................................... *T. marina* (Hirst) comb. nov.- Naso not punctate, spurs of hypostome squat, tarsus II with four solenidia ........................... ...................................................................................................... *T. mortoni* (Luxton) comb. nov.5. Venter with five to six pairs of setae surrounding the genital valve ...................................... 8- Venter with numerous pairs of setae (15 to more than 30 pairs) surrounding the genital  valve ................................................................................................................................................... 66. Venter with >30 pairs of setae; tarsi III and IV with 1–2 and 2–3 solenidia, respectively, ...... ....................................................................................... *T. mexicana* (McDaniel et al.) comb. nov.- Venter with 17–23 pairs of setae; tarsi III and IV with 0 and 1 solenidion, respectively, ....... 77. Venter with 17–20 pairs of setae; seven pairs of genital setae present  .............................................................. *T. hajiqanbari* (Paktinat-Saeij and Kazemi) comb. nov.- Venter with 23 pairs of setae; six pairs of genital setae present .................................................. .................................................................. *T. sebahatae* (Ueckermann and Durucan) comb. nov.8. Setae *c*_2_ almost extending to base of seta *d* ................................................................................. 9- Setae *c*_2_ reaching to over the base of setae *e* or *f* ......................................................................... 109. Prodorsal shield weakly distinct with thin and close longitudinal striations, coxal formula  4-3-4-3 .................................................................. *T. littorina* (Shiba and Furukawa) comb. nov.- Prodorsal shield smooth, distinctly defined and greatly extended reaching upto half of  dorsum, coxal formula 4-6-7-5 ...................................... *T. riccabonai* (Schmölzer) comb. nov.10. Basifemur I with five setae, tibia II nine setae ............................................................................. ........................................ *T. zamaniani* (Khanjani, Asali Fayaz and Ueckermann) comb. nov.- Basifemur I with four setae, tibia II 10 setae ................................................................................... .................................................................... *T. kamalii* (Ueckermann and Khanjani) comb. nov.11. Dorsal setae *c*_1_ and *c*_2_ subequal in length ................................................................................ 12- Dorsal setae *c*_2_ distinctly longer than *c*_1_ ...................................................................................... 1312. Dorsocentral setae *c*_1_ inserted on over extended prodorsal shield ............................................ ...................................................................................................... *P. coineaui* (Judson) comb. nov.- Dorsocentral setae *c*_1_ present on the integument ..... *P. xerophila* (Bernardi et al.) comb. nov.13. All opisthosomal setae on small sclerites ........ *P. aethiopica* (Zmudzinski et al.) comb. nov.- All opisthosomal setae on integument ........................................................................................ 1414. Dorsocentral setae shorter than or equal to the distance between the consecutive setae ....... ........................................................................... *P. hojoensis* (Shiba and Furukawa) comb. nov.- Dorsocentral setae long, crossing the bases of the setae next in line ........................................ 1515. Genu IV with a solenidion ......................................................................................................... 16- Genu IV without a solenidion ....................................................................................................... 1716. Basifemur I with five setae; telofemur III with five setae .. *P. hirsti* (Womersley) comb. nov.- Basifemur I with four setae; telofemur III with four setae ........... *P. leei* (Judson) comb. nov.17. Trochanter IV with 2 setae ........................... *P. khorramabadiensis* (Khanjani et al.) comb. nov.- Trochanter IV with 3 setae ...................................... *P. shiraziensis* (Khanjani et al.) comb. nov.

### 4.6. Additional Notes on the Status of Some Teneriffid Species Excluded from the Key

Among the 28 described species of the family Teneriffiidae so far, six species have incomplete descriptions, insufficient illustrations, and inappropriate species comparisons based on variable morphological characters. These species were excluded from the key and comments have been provided; meanwhile, four species were considered as suggested synonyms due to variable characters used as species diagnosis. These species are as follows.

***Parateneriffia bipectinata* Thor**

*Parateneriffia bipectinata* Thor, 1911:177 [17], McDaniel et al., 1976:532 [10]

The species, *P. bipectinata*, was designated as the type species of the monotypic genus *Parateneriffia*, reported from Paraguay [17]. The original description and illustrations of the species are insufficient, such that important morphological characters for the species differentiation could not be inferred. The author did not illustrate dorsum, gnathosoma, and legs, nor were these body segments described comprehensively. McDaniel et al. [10] provided a very short complementary description and also illustrated only the venter of this species. The most distinct feature provided could be the presence of two transverse sclerotized cleft anterior to the genital slit [10,17]. This character has not been reported since in any of the recently published teneriffid species. Ironically, it now cannot be confirmed as these types of the species have been reported as “lost” [13]. Hence, it was not possible to place it in the diagnostic key provided in the present study.

***Parateneriffia steinbocki* (Irk)** comb. nov. 

*Parateneriffia steinbocki* (Irk) McDaniel et al. 1976:536 [10]

*Mesoteneriffia steinbocki* Irk 1939:222 [21]; Strandtmann, 1965:261 [31]

The monotypic genus *Mesoteneriffia* with its type, *M. steinbocki*, was added in the family Teneriffiidae, by Irk [21] from Ötztal Alps, Austria. The authors provided detailed diagnosis of this genus based on inconsistent (setal arrangement on leg coxae, integument with small pores, absence of genital palps, etc.) and overlapping morphological characters (structure and shape of palp including palp tarsus presence of palp oncophysis, etc.). The type species, *M. steinbocki*, was also insufficiently described and illustrated. 

In the present study, the species *P. steinbocki* comb. nov., is placed in the genus *Parateneriffia* (presence of palponcophysis) and strikingly resembles the species *P. uta* comb. nov., and *P. japonica* comb. nov. It is difficult to discern from later species as leg chaetotaxy, along with other important morphological characters, were not provided in the original description [10,21]. The apparent differences between *P. steinbocki* comb. nov. and *P. uta* comb. nov. could be the length of setae *c*_2_. Ironically, this character cannot be considered as it was found variable between the two different descriptions of *P. uta* comb. nov. [9,31]. The possible differences between *P. steinbocki* comb. nov. and *P. japonica* comb. nov. could be coxal setal formula as 4-4-4-4 vs. 4-3-4-3, respectively. This character in particular is insufficient based on the discussion provided above. Due to morphological similarities and poor descriptions and illustrations, the species, *P. steinbocki* comb. nov. is excluded from the key.

***Parateneriffia alpina* (Schmölzer)** comb. nov.

*Mesoteneriffiola alpina* Schmölzer 1955:36 [22]

The monotypic genus *Mesoteneriffiola* was added in the family based on the collection from “*Unterhalb d. Roche d’Alvau*” [22]. Its species *P. alpina* comb. nov. was designated close to the species *P. steinbocki* comb. nov. and was differentiated from the latter based on the number of coxal setae (Table 1) and position of third pair of prodorsal seta on the prodorsal shield. Similar to *P. steinbocki*, the species *P. alpina* morphologically resembles the species *P. japonica* comb. nov. Although the description and illustration of *P. alpina* comb. nov. are poor, the number of coxal setae are by far the lowest reported in any of the Teneriffid species, i.e., coxae I–IV 3-3-3-1. Other than this, it is difficult to morphologically discern it from the closely related species. 

As a result of new generic divisions proposed in this study, *P. alpina* comb. nov. is placed in the genus *Parateneriffia* but has been excluded from the key due to insufficient morphological description.

***Parateneriffia luxoriensis* (Hirst)** comb. nov.

*Parateneriffia luxoriensis* (Hirst) McDaniel et al. 1976:532 [10]

*Neoteneriffiola luxoriensis* Hirst 1924:1078 [18]

The species *P. luxoriensis* (Hirst) comb. nov. was the type species of the genus *Neoteneriffiola* and is currently placed in the genus *Parateneriffia*. Due to incomplete description, this species is excluded from the key. The closely related species, *P. uta* comb. nov. (later described in 1958) was distinguished based on length of dorsocentral setae and number of setae on palptarsus [31]. Originally, the pedal chaetotaxy and solenidotaxy is neither described nor illustrated [18]. 

***Parateneriffia uta* (Tibbets)** comb. nov.

*Parateneriffia uta* (Tibbets) McDaniel et al. 1976:532 [10]

*Neoteneriffiola uta* Tibbets 1958:44 [24]

This species, *P. uta* (Tibbets) comb. nov., was originally described as closely related to the species *P. luxoriensis* comb. nov. The differential characters used were comparative lengths of dorsocentral setae and number of setae on palp tarsus [24]. The species’ redescription and the key to species provided by Eller and Strandtmann [9] used similar morphological characters. However, McDaniel et al. [10] disagreed with this, stating that inter-setal lengths of dorsocentral setae are variable subject to the state of slide-mounted specimen. Instead, they used the length of leg I vs. body length character in the key. Although the number of setae on palp tarsus was repeatedly used as differential feature, it is unclear if this number in both species includes the solenidion or not [9,10,19,24]. Due to an incomplete description and ambiguity in the diagnostic characters, this species is excluded from the key.

***Parateneriffia tadjikistanica* (Wainstein)** comb. nov.

*Neoteneriffiola tadjikistanica* Wainstein 1969:1250 [12]; Wainstein 1978:202 [32]

*Austroteneriffia tadjikistanica* (Wainstein) Judson 1995:838 [14]

Based on presence of genu palp oncophysis, this species belongs to the genus *Parateneriffia* as proposed in the present study. This species has been reported as morphologically similar to *P. japonica* comb. nov., but this is difficult to discern due to ambiguous leg chaetotaxy [14]. For this reason, the species *P. tadjikistanica* comb. nov. is not included in the presented key.

## 5. Conclusions

Morphological features, which can be used as the generic diagnostic character, must be carefully evaluated. In the family Teneriffiidae, different morphological characters were used over time for generic differentiation, which has led to the unnecessary addition of different genera in the family. In the present research, two genera viz; *Teneriffia* (palpgenus oncophysis absent) and *Parateneriffia* (palpgenus oncophysis present), are recognized in the family Teneriffiidae. This character was found to have been used constantly as one of the generic diagnostic characters since the family Teneriffiidae was recognized [17]. It represents the strength and stability of the character. Through the extensive research performed in the present paper, it is emphasized that such morphological characters must be carefully avoided as they may result in the addition of different genera for a fewer number of species. In contrast, morphological features which provide clear generic differentiations and are persistent even in newly described species must be used. 

## Figures and Tables

**Table 1:** Diagnostic characters of different genera of the family Teneriffiidae.

	*Teneriffia*	*Parateneriffia*	*Neoteneriffiola*	*Heteroteneriffia*	*Austroteneriffia*	*Mesoteneriffia*	*Mesoteneriffiola*	*Sinoteneriffia*	*Himalteneriffia*
Thor [17]	(i) Coxae II not reaching level of coxae III (ii) Oncophysis absent	(i) Coxae II reaching level of coxae IV (ii) Oncophysis present	-	-	-	-	-	-	-
Hirst [18]	(i) Coxae close to each other (ii) Coxae setae numerous (iii) Oncophysis absent	(i) Coxae close to each other (ii) Coxae setae numerous (iii) Oncophysis present	(i) Coxae further apart (ii) Coxae setae less numerous (iii) Oncophysis present	-	-	-	-	-	-
Hirst [19]	-	(i) Coxae close to each other (ii) Oncophysis present (iii) Only leg I–II claw bipectinate (iv) Coxal setae numerous	(i) Coxae further apart (ii) Oncophysis present (iii) Only leg I–II claw bipectinate (iv) Coxal setae less numerous	(i) Coxae further apart (ii) Oncophysis absent (iii) Only leg I claw bipectinate (iv) Coxal setae numerous	-	-	-	-	-
Womersly [20]	(i) Genital disc absent	-	(i) Genital disc absent (based on illustration)	(i) Genital disc absent (based on illustration) (ii) Claws leg I bipectinate (iii) Presence of definite setal row on anterior margin of coxae	(i) Gential disc present (ii) Claws leg I–II bipectinate (iii) Absence of definite setal row on anterior margin of coxae	-	-	-	-
Irk [21]	(i) Oncophysis absent	(i) Oncophysis present (ii) Coxae IV close (iii) Coxae I with backward chitinous process. Before the genital opening a “bracket field”. All four pairs of legs with combs claws.	(i) Oncophysis present (ii) Coxae IV apart	(i) Oncophysis absent	(i) Oncophysis present	(i) Oncophysis present (ii) Coxae IV close (iii) Coxae I without chitinous process. Without “clasp field”. Claws I and II with large and distinct crests, claws of legs III and IV with much smaller ones and only clear at higher magnification	-	-	-
Schmölzer [22]	-	-	-	-	-	(i) Coxal setae 4-4-4-4	(i) Coxal setae 3-3-3-1	-	-
McDaniel et al. [10]	(i) Numerous ventral opisthosomal setae (ii) Palpal genu distal process absent (iii) Lengths subequal to or shorter than body length (idiosoma + gnathosoma); each coxa with 4 or more setae arranged along anterior margin and 1 longer seta on posterior margin	(i) Few ventral opisthosomal setae (ii) Palpal genu with or without a distal process (iii) Lengths subequal or longer than body (including gnathosoma): coxae with 7 or fewer setae, usually 3 or 4; anterior tarsal claws strongly bipectinate; posterior tarsal claws simple or weakly bipectinate, with empodial claws	Synonymized				Not Mentioned	-	-
Yin et al. [23] (did not consider McDaniel et al. [10])	-	-						(i) 1 dorsal plate on the forefoot body and no plate on the back of the hind body (ii) Suckers and no setae on the genital valve (iii) Coxae 4-3-4-2 (iv) 2 pairs of setae on both sides of the reproductive valve	-
Judson [13]	-	-	Reinstated: (i) Called work of McDaniel premature (ii) The strong, linear neotrichy of the pedal solenidia (iii) The relatively large dorsal plates of the opisthosoma (iv) The reduced form of the peritremes	Remained Synonymized				-	-
Judson [14]	-	-	-	Remained Synonymized	Reinstated: With detailed diagnosis but no comparison with other genera. He added many characters in the original diagnosis, important as are commented	Remained Synonymized		-	-
Schmölzer [15]	-	-	-		-			-	1. Idiosoma dorsal with a structureless one that reaches almost half the length of the idiosoma Dorsal field. 2. Claw ridges strong on the first two pairs of legs, on legs III and IV still clear, but becoming weaker and weaker. 3. Number of coxal bristles on legs I–IV according to the scheme 4-6-7-5.

**Table 2:** Chronological information for the genera and species in the family Teneriffiidae.

**(a) Species and the genera they previously belonged to**		
**Genus (as reported in the literature)**	**Species**	**Year**
*Teneriffia*	*quadripapillata*	Thor [17]
*Parateneriffia*	*bipectinata*	Thor [17]
*Neoteneriffiola*	*luxoriensis*	Hirst [18]
*Heteroteneriffia*	*marina*	Hirst [19]
*Austroteneriffia*	*hirsti*	Womersley [20]
*Mesoteneriffia*	*steinbocki*	Irk [21]
*Mesoteneriffiola*	*alpina*	Schmölzer [22]
*Neoteneriffiola*	*uta*	Tibbets [24]
*Austroteneriffia*	*japonica*	(Ehara [11])
*Austroteneriffia*	*tadjikistanica*	(Wainstein [12])
*Austroteneriffia*	*hojoensis*	(Shiba and Furukawa [5])
*Austroteneriffia*	*littorina*	(Shiba and Furukawa [5])
*Teneriffia*	*mexicana*	McDaniel et al. [10]
*Teneriffia*	*mortoni*	(Luxton [25])
*Neoteneriffiola*	*coineaui*	Judson [13]
*Sinoteneriffia*	*nuda*	Yin et al. [23]
*Austroteneriffia*	*leei*	Judson [14]
*Sinoteneriffia*	*kunmingensis*	Youzhen et al. [26]
*Neoteneriffiola*	*yunnanensis*	Youzhen et al. [27]
*Austroteneriffia*	*kamalii*	Ueckermann and Khanjani [7]
*Himalteneriffia*	*riccabonai*	Schmölzer [15]
*Austroteneriffia*	*zamaniani*	Khanjani et al. [28]
*Neoteneriffiola*	*xerophila*	Bernardi et al. [1]
*Austroteneriffia*	*shiraziensis*	Khanjani et al. [29]
*Austroteneriffia*	*khorramabadiensis*	Khanjani et al. [30]
*Teneriffia*	*sebahatae*	Ueckermann and Durucan [7]
*Teneriffia*	*aethiopica*	Zmudzinski et al. [2]
*Teneriffia*	*hajiqanbari*	Paktinat-Saeij and Kazemi [8]
**(b) Number of genera and species previously added over time**		
**Year**	**Number of Genera**	**Number of Species**
1911	2	2
1924	3	3
1925	4	4
1935	5	5
1939	6	6
1955	7	7
1958	7	8
1965	7	10
1969	7	11
1975	7	12
1976	2	13
1993	2	14
1994	3	16
1995	4	17
1996	4	18
1997	5	19
2002	6	21
2011	6	22
2012	6	23
2013	6	24
2014	6	25
2020	6	26
2021	6	27
2022	6	28

**Table 3:** Diagnostic characters of two genera proposed in this study, their species and distribution.

Species	Genus	Distribution	Year	Species Characters						
				Prodorsal Shield	Dorsal Setae	Ventral Setae around G	Tarsi III–IV	Coxae I–IV	Genu Oncophysis	Length of c2
*mexicana*	*Teneriffia* Thor	Mexico	1976	present	on cuticle	multiples	divided	7/10-7/12-7/10-6/9	absent	crossing d
*quadripapillata*		Spain	1911	present	on sclerite	15 pairs	undivided	7-8-6-6	absent	crossing d
*sebahatae*		Turkey	2020	present	on cuticle	23 pairs	divided IV	7-8-6-6	absent	reaching d
*hajiqanbari*		Iran	2022	present	on cuticle	17–20 pairs	divided	6/7/8-6/7-6/7-5	absent	reaching d
*kamalii*		Iran	2002	present	on cuticle	6 pairs	not described	4-3-4-3	absent	reaching f
*zamaniani*		Iran	2011	present	on cuticle	6 pairs	divided	4-3-4-3	absent	reaching e
*littorina*		Japan	1975	inconspicuous	on cuticle	6 pairs	not described	4-3-4-3	absent	reaching d
*riccabonai*		India	2002	present	on cuticle	5 pairs	divided	4-6-7-5	absent	subequal to all
*marina*		Japan, Malaysia	1925	absent	on cuticle	more than 30 pairs	divided	6/7-7/10-7/8-5/8	absent	subequal to all
*mortoni*		Japan	1993	absent	on cuticle	atleast 40	divided	8-7/8-8/9-8	absent	reaching d
*aethiopica*	*Parateneriffia* Thor	Ethiopia	2021	present	on sclerite	6–7 pairs	divided	7-6-6-5	present	reaching e
*coineaui*		Namibia	1994	present	on sclerite	5 pairs	divided	4-3-4-3	present	subequal to all
*xerophila*		Brazil	2012	present	on sclerite	5 pairs	not described	3-4(6)-4-3	present	subequal to all
*uta*		Mexico, USA	1958	present	on cuticle	5 pairs	divided	4-3-4-3	present	reaching f
*hojoensis*		Japan	1975	present	on cuticle	6 pairs	divided	4-3-4-3	present	crossing d
*hirsti*		Australia	1935	present	on cuticle	6 pairs	divided	4-3-4-3	present	crossing d
*khorramabadiensis*		Iran	2014	present	on cuticle	6 pairs	divided	4-3-4-3	present	crossing h
*shiraziensis*		Iran	2013	present	on cuticle	6 pairs	divided	4-3-4-3	present	reaching h
*leei*		Australia	1995	present	on cuticle	6 pairs	divided	4-3-4-3	present	not described
*bipectinata*		Paraguay	1911	not described	not described	not described	not described	3-3-7-4	present	not described
*steinbocki*		Austria, Switzerland	1939	present	on cuticle	not described	divided	4-4-4-4	present	reaching d
*alpina*		France	1955	present	on cuticle	4 pairs	divied	3-3-3-1	present	reaching e
*tadjikistanica*		Tadjikistan, Yemen	1969	present	on cuticle	6 pairs	divided	4-3-4-3	present	longer than other
*luxoriensis*		Egypt	1924	inconspicuous	on cuticle	5–6 pairs	divided	4-3-4-3	present	reaching d

**Table 4:** List of species in the family Teneriffiidae and their developmental stages (the green color represents the stage/s described).

Genus	Species	Larva	Nymph			Adult	
			Proto-	Deuto-	Trito-	Male	Female
*Teneriffia*	*quadripapillata*						
*Parateneriffia*	*bipectinata*						
*Neoteneriffiola*	*luxoriensis*						
*Heteroteneriffia*	*marina*						
*Austroteneriffia*	*hirsti*						
*Mesoteneriffia*	*steinbocki*						
*Mesoteneriffiola*	*alpina*						
*Neoteneriffiola*	*uta*						
*Austroteneriffia*	*japonica*						
*Austroteneriffia*	*tadjikistanica*						
*Austroteneriffia*	*hojoensis*						
*Austroteneriffia*	*littorina*						
*Teneriffia*	*mexicana*						
*Heteroteneriffia*	*mortoni*						
*Neoteneriffiola*	*coineaui*						
*Sinoteneriffia*	*nuda*						
*Austroteneriffia*	*leei*						
*Sinoteneriffia*	*kunmingensis*						
*Neoteneriffiola*	*yunnanensis*						
*Austroteneriffia*	*kamalii*						
*Himalteneriffia*	*riccabonai*						
*Austroteneriffia*	*zamaniani*						
*Neoteneriffiola*	*xerophila*						
*Austroteneriffia*	*shiraziensis*						
*Austroteneriffia*	*khorramabadiensis*						
*Teneriffia*	*sebahatae*						
*Teneriffia*	*aethiopica*						
*Teneriffia*	*hajiqanbari*						

## Data Availability

All necessary data for the manuscript is provided.
